# Fish-eye camera and image processing for commanding a solar tracker

**DOI:** 10.1016/j.heliyon.2019.e01398

**Published:** 2019-03-29

**Authors:** Gerardo Garcia-Gil, Juan M. Ramirez

**Affiliations:** a Electrical Engineering Department, Centro de Enseñanza Técnica Industrial, Calle Nueva Escocia 1885, Providencia 5a Sección, Guadalajara, 44638, Jalisco, Mexico; b Electrical Engineering Department, Cinvestav del IPN, Av. del Bosque 1145, Col. El Bajío, Zapopan, 45019, Jalisco, Mexico

**Keywords:** Engineering, Electrical engineering

## Abstract

The design and implementation of a solar tracker based on panoramic images captured by a fisheye camera are proposed. Such images receive a digital treatment to estimate the sun azimuth and the elevation angles. These angles are fed to a microcontroller, handling an accelerometer with a gyroscope, that positions the solar tracker to the angle of solar elevation and a compass to set the azimuth angle concerning the north, either magnetic or geographic. The proposed system works satisfactorily to guide the solar tracker regardless of whether it is a sunny or cloudy day.

## 1 Introduction

Development of technologies that allow the efficient use of solar energy as a source of renewable energy has boomed in recent decades. For this reason, systems dedicated to capturing and using this energy to produce electricity, heat or light are continually improved, ensuring that their operation is as efficient and economical as possible. One of the best-known methods for this purpose is the use of photovoltaic panels to generate electricity from solar radiation. However, despite diverse improvements, the efficiency of these devices is still low. There are several factors causing reductions in efficiency, including temperature, the cell's quality, the radiation received in the region and the incidence angle in which solar radiation is received. The surface of the photovoltaic system PV is limited, and therefore it is desirable to be optimally utilized, receiving as much radiation as possible on it. In this paper, the problem of positioning the solar-thermal collector is addressed. This aspect has been of great importance for the improvement of efficiency, since the amount of electrical energy that a photovoltaic panel can produce, strongly depends on the position in which it receives solar radiation [1]. A good position for the photovoltaic system PV should be free of obstructions blocking sunlight and should meet that condition throughout the day. The fraction of total energy emitted by the sun that reaches the surface of the solar cell is directly related to the position in which it is located [2]. Thus, the detection of the sun's position and the construction of devices able to guide the PV panel so that it captures the greatest amount of sunlight, plays a fundamental role in the use of solar energy. By placing the panels in the direction of the sun's rays, the radiation they can capture is maximized; the improvement in efficiency is significant concerning a static cell [3]. The device used to carry out this task is called a solar tracker and allows you to track the sun's position and follow it. There are several models of solar trackers, with different types of operation.

A recently published reference proposes an image treatment similar to the one proposed here, with a higher computational cost [4]. The irradiance is assessed based on the shape's contours and does not use a universally accepted comparison of results. Calculate the irradiance every 20 minutes, and there is no demonstration of the angle estimation's precision.

The design of solar trackers began when there were significant improvements in energy production compared to static panels; for instance, Abderrahmane published in 1990 that an increment of up to 40% in the production of energy is attained [5]. In 1975, McFee presented the first solar tracker to perform its task automatically [6], where a description of the analog operation system that has the facility to be used to measure the radiation received by a solar panel.

Solar trackers may be classified according to their number of axes as described in the following section [7].

## 2 Background

Onyeka wrote the available solar potential of some selected locations in Nigeria on an inclined single-axis tracker (considering six tracking orientations) and dual-axis tracking surfaces are assessed using hourly radiation data [8]. Bahrami presents the effect of latitude on the performance of different solar trackers. The hourly solar radiation data of different locations around Europe and Africa measured on a horizontal surface is collected and utilized [9]. The author indicates that energy produced from a typical PV panel with or without a solar tracker is mainly dependent on the available solar irradiance. Interestingly, for some locations on nearly the same latitude in the northern hemisphere, the solar irradiance varies significantly and this aspect must be taken into account in solar trackers. For this reason, the present study aims to explore the effect of solar irradiation on the performance of PV panels incorporating a solar tracker [10].

### 2.1 Dual-axis

Dual-axis trackers have two degrees of freedom functioning as axes of rotation. Commonly these axes are arranged orthogonally to each other. Usually, the primary axis is fixed to its base while the second axis is generally arranged orthogonally to it. There are several configurations for this type of tracker, classified by the orientation of their primary axis concerning the ground. Two of the most common configurations become: (*i*) double-axis inclined; and (*ii*) oriented respect to the azimuth-altitude [11]. Also, considering the technology used to track the solar position, three groups of solar trackers may be found.

### 2.2 Active dual-axis

These trackers use photo sensors and electronic systems to control motors in charge of producing the tracker's movement. The most widely used type is variable resistors LDRs (Light Dependent Resistor) [12], whose resistance value varies depending on the amount of incident radiation. A real-time system monitors the prevailing lighting conditions by providing constant feedback and reacting according to the acquired data [6]. The reliability of these devices is limited since their electrical parts are sensitive to the climate, as well as quite prone to damage when there are lightning discharges. Since its operation depends on electronic sensors and these devices are easily obstructed, the tracker can have serious conflicts and seriously hamper the energy production, so it is necessary to monitor them continuously.

### 2.3 Chronological tracker

Such tracker is a programmed system that uses astronomical knowledge and geographical coordinates to determine the sun position every day. This type of tracker does not acquire lighting information because it works similarly to a clock, with a defined trajectory throughout the day. Usually, it is controlled by complicated programmed electronic systems, or it can work simply with an engine that moves it 15° every hour, generating either a complete or partial rotation. Its control system is complex, and in some cases, it may require computer systems in permanent operation. Just as active trackers, they are quite vulnerable to the environment and electric discharges. It has great advantages when precision is important, as well as when used in distant latitudes to the equator where winters are intense [13].

### 2.4 Passive tracker

This type of tracker does not use an external energy source, but employs thermal energy from the sun's rays, converting it into mechanical energy, using elements with adequate thermal properties to perform such tasks. Passive devices use basic physical principles to collect heat instead of using electromechanical devices. The heat collected is used by placing the elements conveniently. The accuracy of this type of trackers, in general, is not high; however, due to their cost and easy maintenance, they are quite affordable. These systems use lateral shock absorbers to prevent sudden movements caused by the wind or other factors so that the movement of the tracker is directed by a system of counterweights [14].

Other trackers have been documented. For example, there is some that use Fresnel lenses on the solar panels' surface. However, being a method that introduces a layer of material over the cells, they absorb a certain amount of the radiation, as well as reflect another amount of it [15]. Also, a solar tracker with a fast digital system on board with a mobile platform in motion has been built and implemented. The tracker consists of two rotating mirrors and one lens [16]. Moreover, proper software can calculate the circadian sun orbit and consequently to move the solar panels to maintain the panel's surface perpendicular to solar rays, improving the efficiency in energy production [17]. The main contributions of this paper are as follows:

▪ Use of a fisheye camera as the only input sensor for tracking.
▪ A simple and reliable algorithm is proposed.
▪ Photo-resistances and a Global Positioning System (GPS) are not required.
▪ Provision of a fast robust and truthful system for the sun's location.
▪ Only one fisheye camera is required to position several solar trackers.

## 3 Methodology

**Fig. 1:**
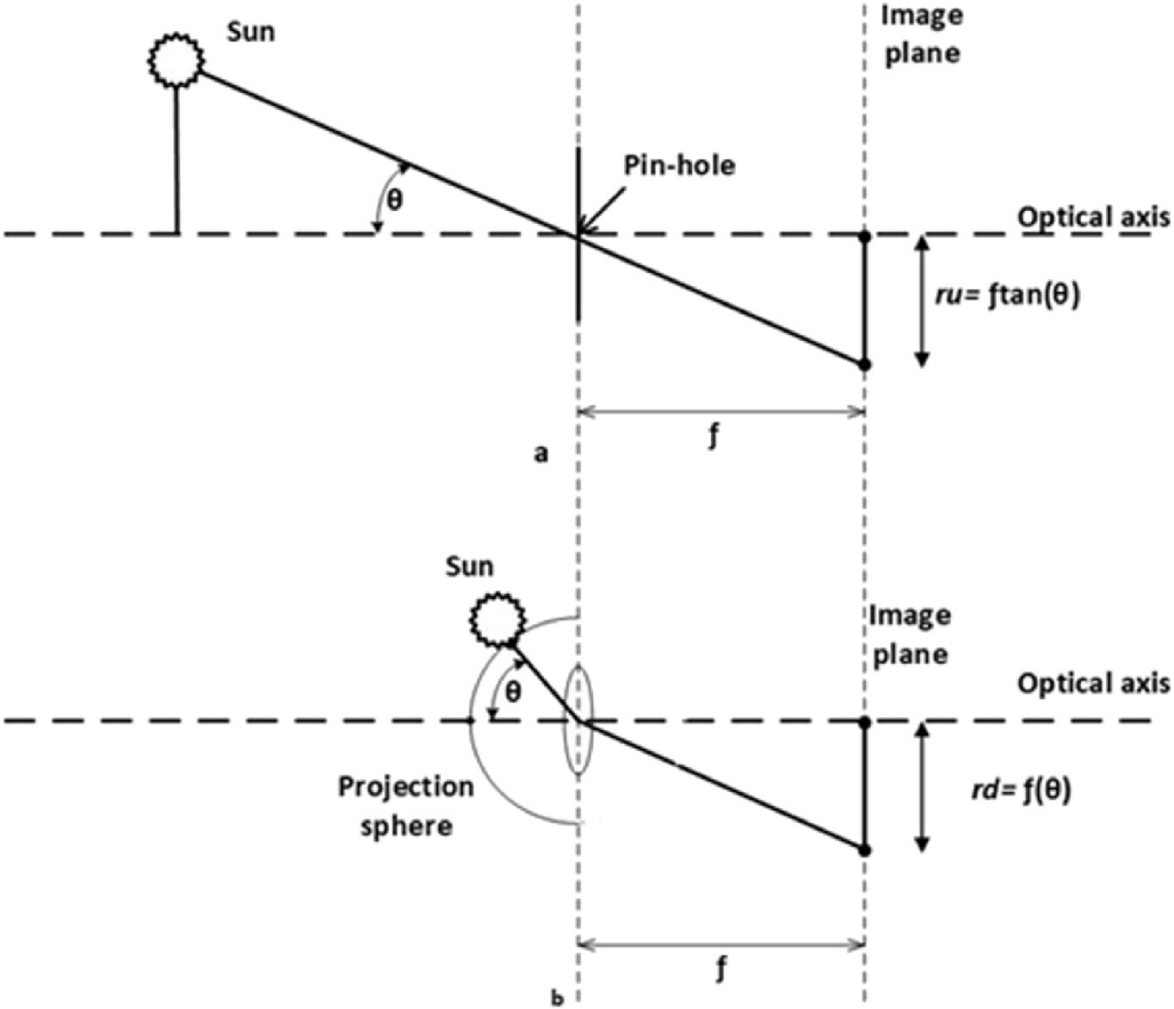
The rectilinear pinhole and equidistance fish-eye projections.

It is noteworthy to mention that the azimuth and elevation are the two coordinates that define the position of a celestial body (sun) in the sky as viewed from a particular location at a particular time. Notice that the fisheye camera has been leveled and oriented toward the North; this is of great importance for the geo-solar calculation in this proposition [18]. In this paper, the images are taken from the fisheye camera *ALLSKY 340C*. The proposed solar tracker bases its functioning on such images with angles of view to 180°, which receive a digital treatment before the azimuth and solar elevation angles can be inferred. Then, the position of the PV is controlled by the solar tracking system to maximize the amount of solar energy regardless of the sun's position. The equidistance projection model is the most commonly used by fish-eye cameras; the fish-eye and pinhole camera projections are depicted in Fig. 1. The perspective projection made by a pinhole camera as given in (1),

(1) $$r_{u}=ftan\left(\theta \right)$$

where $r_{u}$ is the undistorted distance of the projected point from the centre, and θ is the angle that the projected point makes with the optical axis at the centre of projection. The centre of distortion is assumed to be the centre of the image; *f* is the focal distance.

On the other hand, fish-eye lenses are usually designed to obey one of the following projections: (*i*) stereographic projection; (*ii*) orthogonal angle projection; (*iii*) equisolid angle projection; (*iv*) equidistance projection.

Respect to the equidistance with a fish-eye camera [19], it may be estimated by (2),

(2) $$r_{d}=f\left(\theta \right)$$

where $r_{d}$ is the distorted height of the projected point on the image plane. Managing these two Eqs. (3) and (4) the conversion is obtained from equidistant image to the rectilinear image plane,

(3) $$\;\;r_{u}=ftan\left(\frac{r_{d}}{f}\;\right)$$

and

(4) $$\;\;\;r_{d}=ftan^{-1}\left(\frac{r_{u}}{f}\;\right)$$

### 3.1 Grayscale conversion

**Fig. 2:**
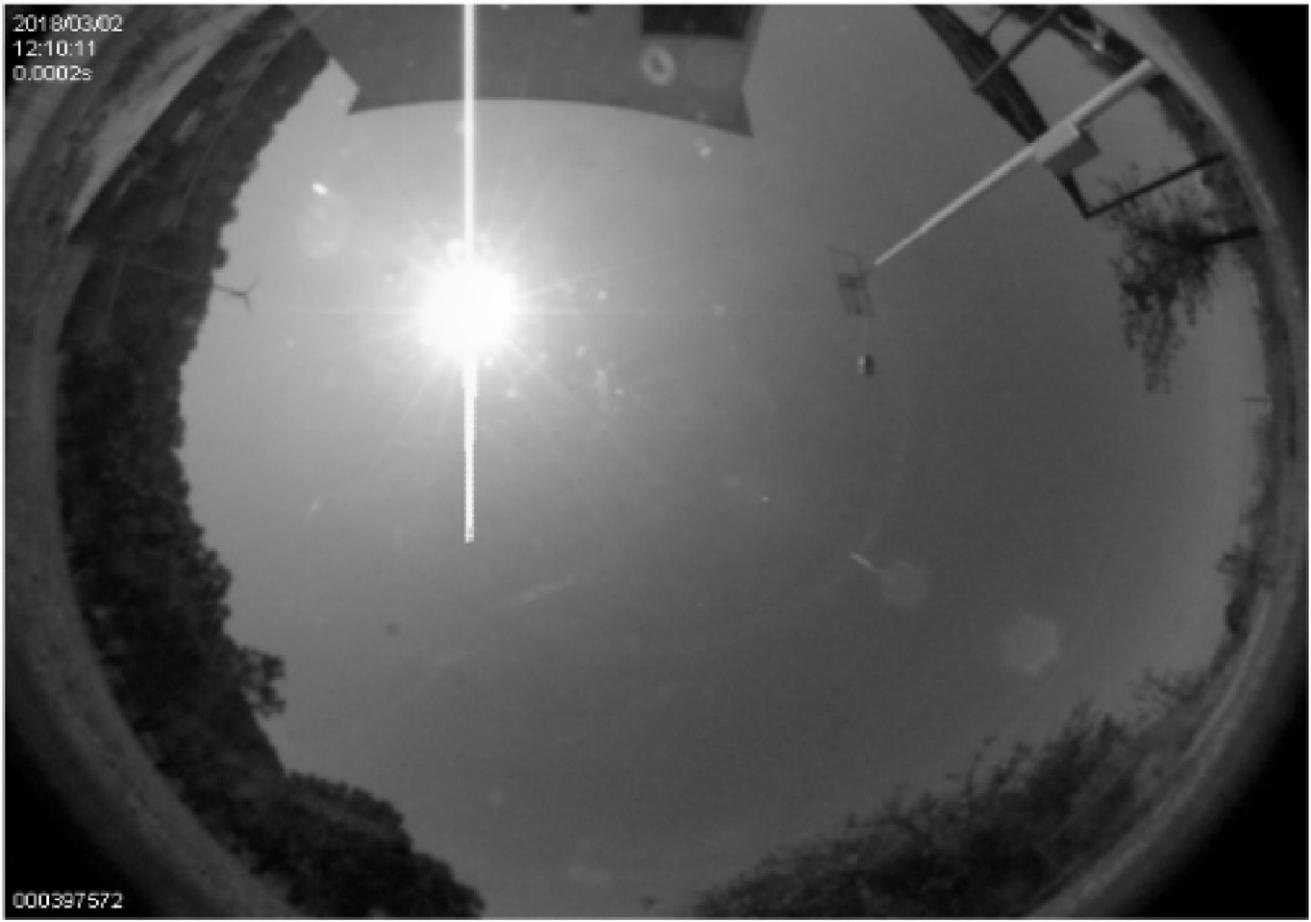
Grayscale image.

The simplest algorithm for obtaining a grayscale is that of the average, which consists of calculating the average of the RGB channels. For example, given a matrix of dimension MxNx3 corresponding to an image, the pixel of the grayscale matrix for the (*i, j*)-position is calculated in (5),

(5) $$XG_{i,j}=\frac{X_{i,j,1}+X_{i,j,2}+X_{i,j,3}}{3}$$

where *XG* becomes the grayscale matrix of dimension MxN. X_i, j, 1_, X_i, j, 2_ and X_i, j, 3,_ are the corresponding *R, G,* and *B* channels [20, 21, 22, 23]. Fig. 2 illustrates the result of applying Eq. (5) to obtain the grayscale image. It is noteworthy that this strategy allows to work with 256 gray-scale and not 256^3^ colors used in a conventional RGB scheme, computationally more expensive.

### 3.2 Segmentation by threshold

**Fig. 3:**
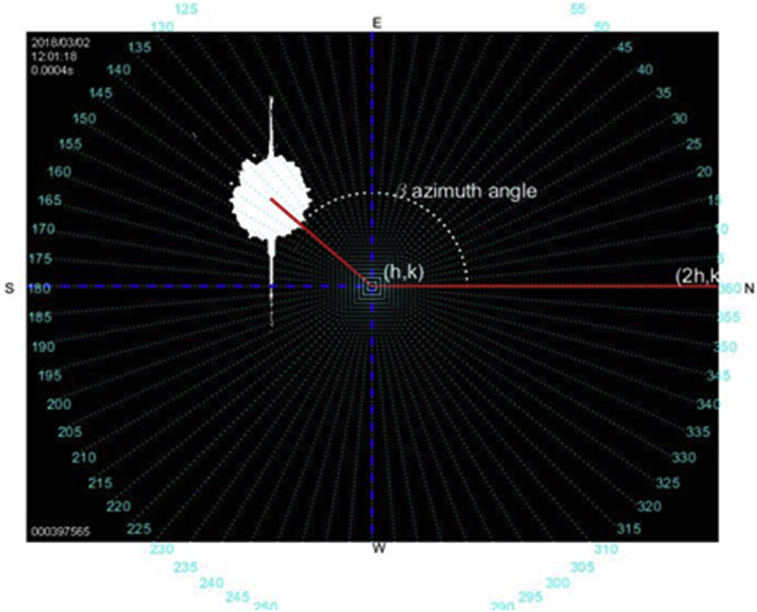
Binary image obtained with the fisheye cam; center at (*h, k*) pixels.

The segmentation by threshold may be considered as a special form of quantification in which the pixels of the image are divided into two classes [21], depending on a predefined threshold (*P*_*th*_); in this application, *P*_*th*_ is 240. All the images's pixels take two different values P_0_ or P_1_, depending on the relation they keep with the threshold, formally defined as given in (6),

(6) $$f_{th}\left(P\right)=\{\begin{array}{c} P_{0}\;if\;P<P_{th}\\ P_{1}\;if\;P\geq \;P_{th} \end{array}$$

where 0 < P_th_ < P_max_, P_max_ = 256. This operation results in the binarization of a grayscale image shown in Fig. 2, assuming P_0_ = 0 and P_1_ = 1, once it has been binarized through Eq. (6). Notice that segmentation and 256 scale-of-grays are used [24, 25]. That is, in this paper 256 grays are used, where the black tone (absence of all color) is associated to P = 0, and the white tone (presence of full color) is associated by P = 256. Then, the image's binarization takes P = 240, where the values greater than this will be white P = 1, and the lower ones will be black P = 0, Fig. 3.

A digital image processing is carried out before the calculation of the required angles to guide the solar tracker. To estimate the centroid of the image's brightest portion the following procedure is carried out, Fig. 3.

The image becomes a succession of pixels, where the center (*h, k*) and radius *r* may be estimated. The center is estimated by dividing the length (in pixels) of the horizontal and vertical axes by 2. Then, the center of the circle is located on the figure (*h, k*), and a horizontal reference line is chosen toward the North. The next step consists of the centroid's estimation of the brightest object within the image [22]. This process is quite important because the object's centroid must be precisely estimated, which is one of the relevant advantages in the proposed method. That is, placing the solar tracker at the point where it receives the highest irradiance, either from the sun or some flash in some cloud near to this one. Eqs. (7) and (8) are used to estimate the centroid of the most enlightened body resulting from the binary image, Fig. 3.

(7) $$X_{c}=\frac{\sum_{i=1}^{n}{\bar{x}}_{i}A_{i}}{\sum_{i=1}^{n}dA}=\frac{\int xdA}{\int dA}$$

(8) $$Y_{c}=\frac{\sum_{i=1}^{n}{\bar{y}}_{i}A_{i}}{\sum_{i=1}^{n}dA}=\frac{\int ydA}{\int dA}$$

where *X*_*c*_ and *Y*_*c*_ are used in Eq. (12) and is important since it is a component of one of the vectors to calculate the azimuth and the elevation angles, respectively.

The above coordinates are transformed to the 2D plane of the image using the *f-theta system of the lens* [26], found to provide a good initial fit. Then, they are shifted and rotated to account for the fact that the camera does not point precisely to the zenith, nor is the image top perfectly aligned with the true north. Finally, an empirical scaling factor is applied to the coordinates in the *h* and *k* direction to compensate for the distortion created by the lens [18]. Such transformation represents a difference between the sun path follows by the national oceanographic and atmospheric administration of the United States (NOAA) [27], and the proposed solar tracking. The errors as a function of the *x*, and then *y* directions are fitted to a bi-cubic function and are added to the coordinates are given in (9), (10),

(9) x=τzsin(A−Δ)+h

(10) y=τzcos(A−Δ)+k

where *z* stands for the vertical angular distance between the sun and the observer's local horizon or the observer's local plane. The elevation of the sun is the angle between the direction of the geometric center of the sun's apparent disk and the observer's local horizon; *A* stands for the azimuth angle (angle between the sun and the North, measured clockwise around the observer's horizon); Δ stands for the camera's rotation from the North (it may take into account some imperfections of the mechanism and possible de-calibration of the equipment due to its mobility). For the fish-eye cameras (*h, k*) τ is a scale factor; in this paper it is taken as 3.365 px/deg [18]. The above expressions as given in (11),

(11) $$\tau z=\sqrt{{{\left(x-h\right)}^{2}+\left(y-k\right)}^{2}}$$

**Fig. 4:**
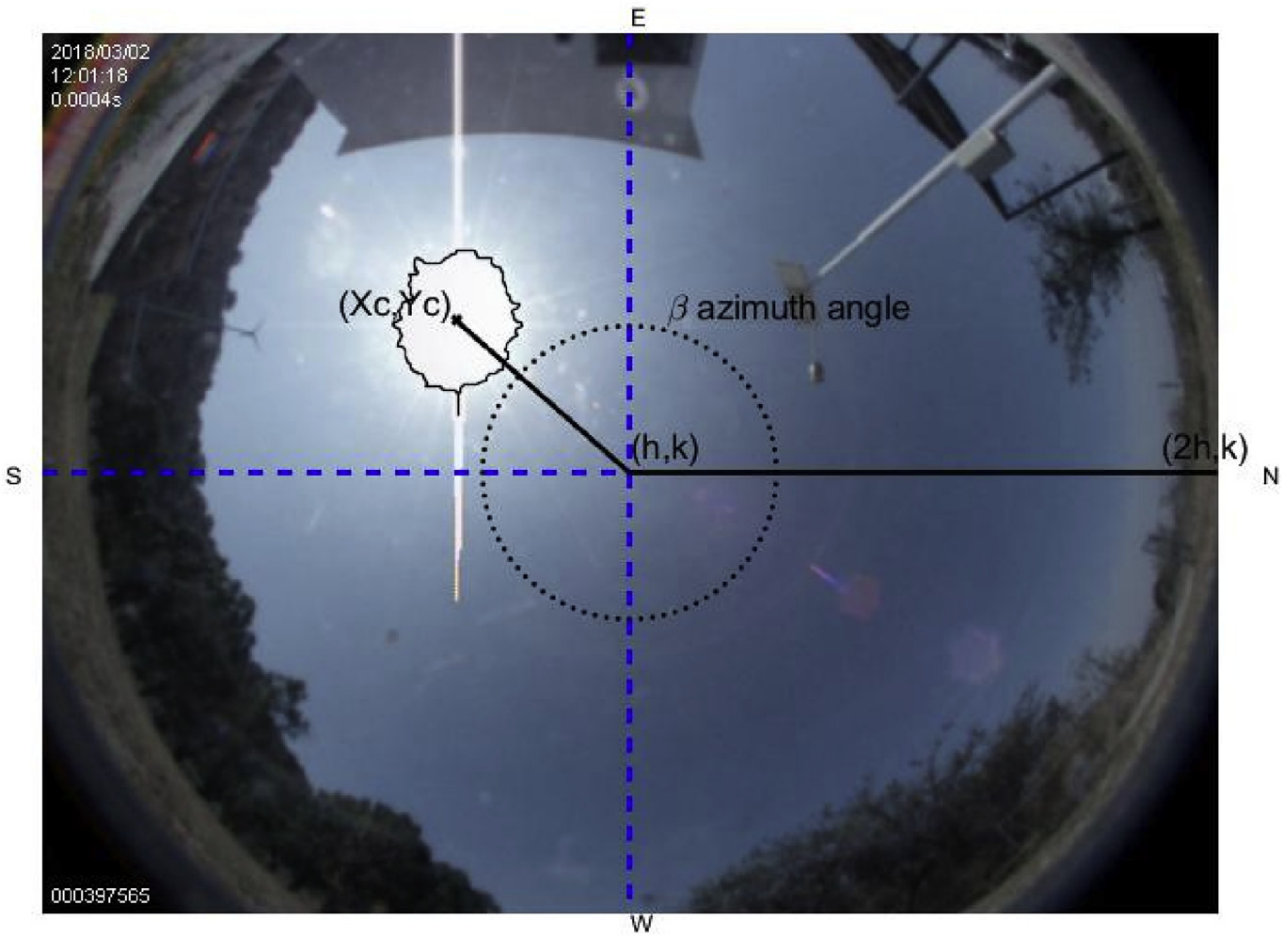
Segments to calculate the azimuth and the solar elevation angles.

The result was found to align well in the central parts of the image, and slightly less well around the edges. The Eqs. (12) and (13) which map astronomic coordinates into the camera ones are,

(12) $$\begin{array}{l} d=\sqrt{{{\left(X_{c}-h\right)}^{2}+\left(Y_{c}-k\right)}^{2}} \end{array}$$

(13) $$\begin{array}{l} e=h=\sqrt{{{\left(2h-h\right)}^{2}+\left(k-k\right)}^{2}} \end{array}$$

where *d* is the distance from the center of the image to the centroid of the sun*, e* is the distance from North to the center of the image; Fig. 4 exhibits the vector resulting from the application of Eqs. (12) and (13) to the centroid, using the expression of the distance between two points. The calculation of the azimuth angle is obtained as given in (14),

(14) $$\beta =\Delta +tan^{-1}\left[\frac{m_{2}-m_{1}}{1+m_{1}m_{2}}\right]$$

where Δ is the corresponding displacement of the camera relative to the north given in degrees; $\;m_{1}\;$and $m_{2}$ are the line's slopes. One-line results of joining the center (*h, k*) with the point (*Xc, Yc*), the second line arises by joining the center of the image (*h, k*) to the image's north orientation (*2h, k*). Fig. 4 displays the azimuth angle β. For the solar elevation angle calculation, Eq. (15) can be used. The cosine of angle α coincides (except in sign) with the cosine of the angle formed by the line's director vectors *d – e*, the scalar projection of a vector *d* on a vector *e*, also known as the scalar resolute of *d* in the direction of *e*, is given by:

(15) $$d=\left|e\right|\;\cos\;\alpha \;,\;\alpha =\cos^{-1}\left(\frac{d}{\left|e\right|}\right)$$

**Fig. 5:**
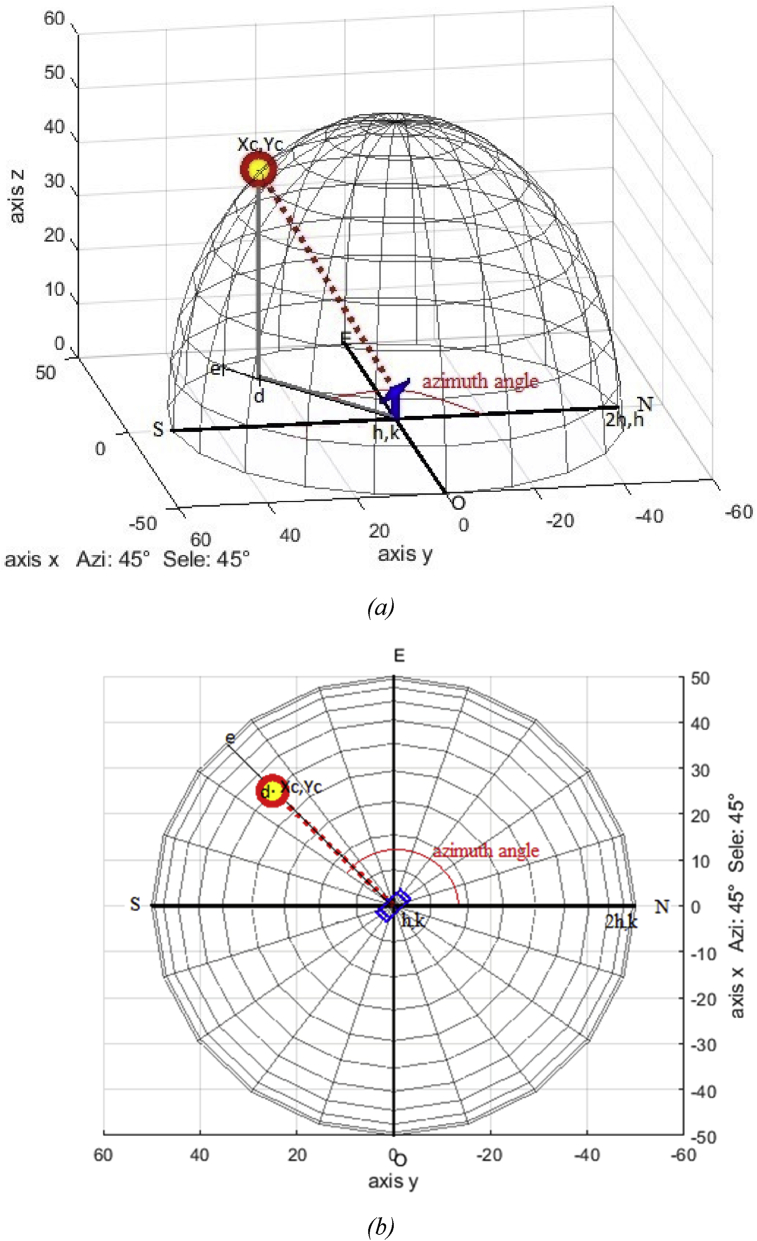
(*a*) Solar elevation angle at 45°, (*b*) Sun azimuth at 45°.

Alternatively, the cos(α) may be computed in terms of the dot product between *d* and *e*, that is the calculation of the elevation angle. The elevation angle is obtained after having calculated the sun azimuth angle as given in (16); its graphic representation is shown in Figs. 5a and 5b,

(16) $$\cos\;\alpha =\left(\frac{\left|\vec{u}\cdot \vec{v}\right|}{\left|\vec{u}||\vec{v}\right|}\right)=\frac{\left|u_{1}v_{1}+u_{2}v_{2}\right|}{\sqrt{u_{1}^{2}+u_{2}^{2}}\sqrt{v_{1}^{2}+v_{2}^{2}}},\;\alpha \in \left[0,\frac{\pi }{2}\right]$$

where $\vec{u}\;$ and $\vec{v}$ stand for the director vectors of lines *d* and *e*, that is, $\vec{u}\;$ = ($u_{1},u_{2}$) and $\vec{v}$ = ($v_{1},v_{2}$). Then, the solar elevation angle is obtained after having calculated the azimuth angle, Fig. 5.

The above images come from the simulation based on Matlab, where the solar position in the sun azimuth and the solar elevation angle have been obtained from (NOAA) [26]. Fig. 5 illustrates the sun azimuth and the elevation angles for 45°, which inspires the idea of this solar tracker. The sun azimuth and elevation angles can be estimated without requiring calibrating and installing more instruments, nor performing complex calculations as in other methodologies [28, 29, 30, 31, 32].

The simplicity of the proposed algorithm lies in the estimation of the sun azimuth and elevation angles that are compared with the readings obtained instantly from the accelerometer and magnetometer installed at the Dual-Axis that will move until the angles obtained from devices and the algorithm become equal. Algorithm 1 summarizes the proposed algorithm.

**Table:** 

**Algorithm 1**: Solar tracker algorithm
1. **Initialization:** Take an image from fisheye cam 2. **Operation;** Calculate the azimuth angle & solar elevation angle obtained from processing digital images Get the current measurements of the accelerometer and the compass 3. **Main loop:** Begin repeat **If** azc* = azm Stop azimuth motor If aesc* = aesm Stop solar elevation motor **Else if** aesc* < aesm High solar elevation motor **Else** Low solar elevation motor **End if** Else if azc* < azm Left azimuth motor **Else** Right azimuth motor **End if** 4. The program is finished, but if it does not the algorithm is started in step 3. Notice that azc* is the calculated azimuth angle; azm stands for the measured azimuth angle; aesc* is the calculated solar elevation angle; and aesm means the measured solar elevation angle.

## 4 Experimental

### 4.1 Study case March 2, 2018

**Fig. 6:**
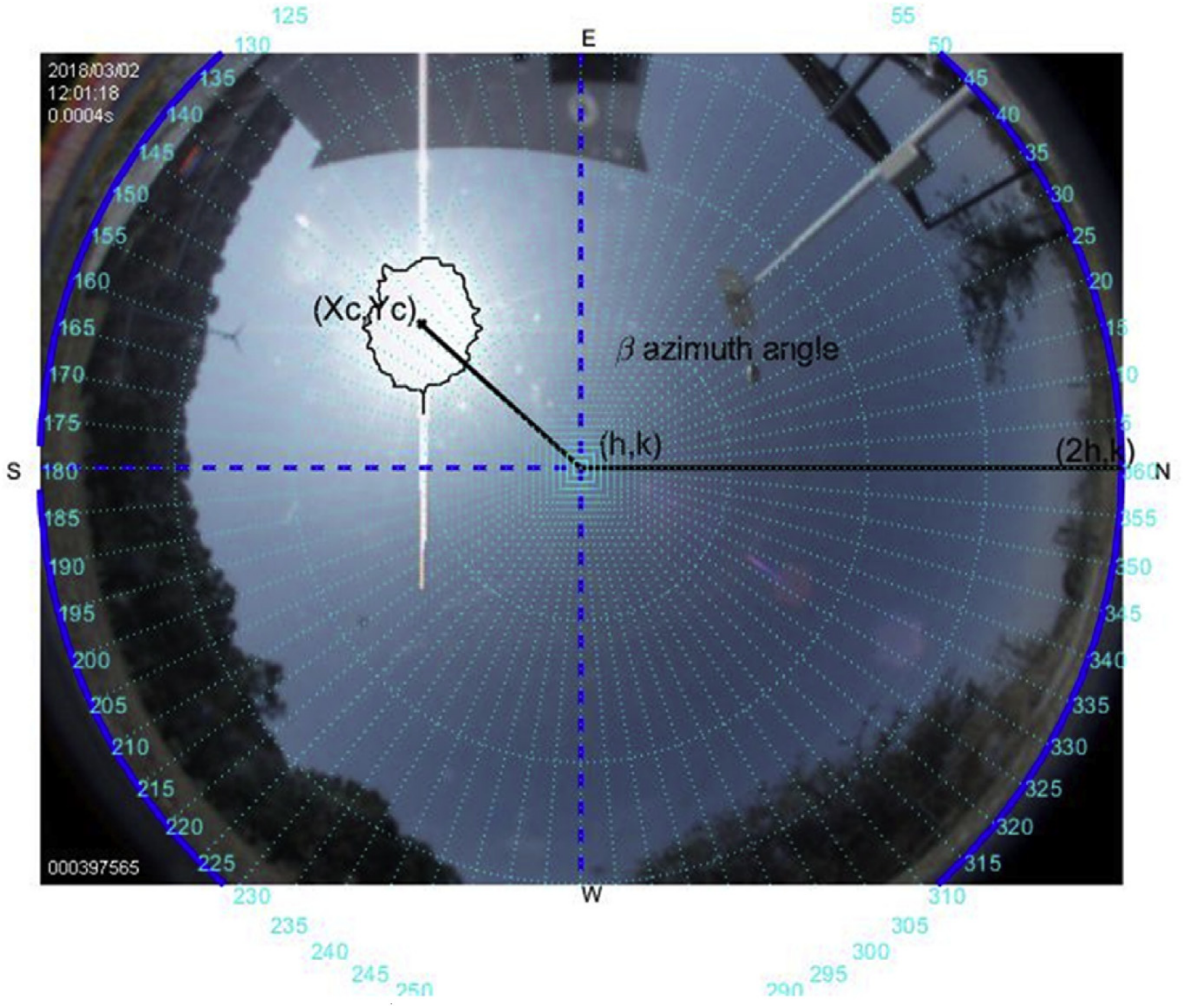
The image was taken at 12:00 hrs on March 2, 2018; the sun azimuth angle is 148.32° degrees and the solar elevation angle, 55.28°.

**Fig. 7:**
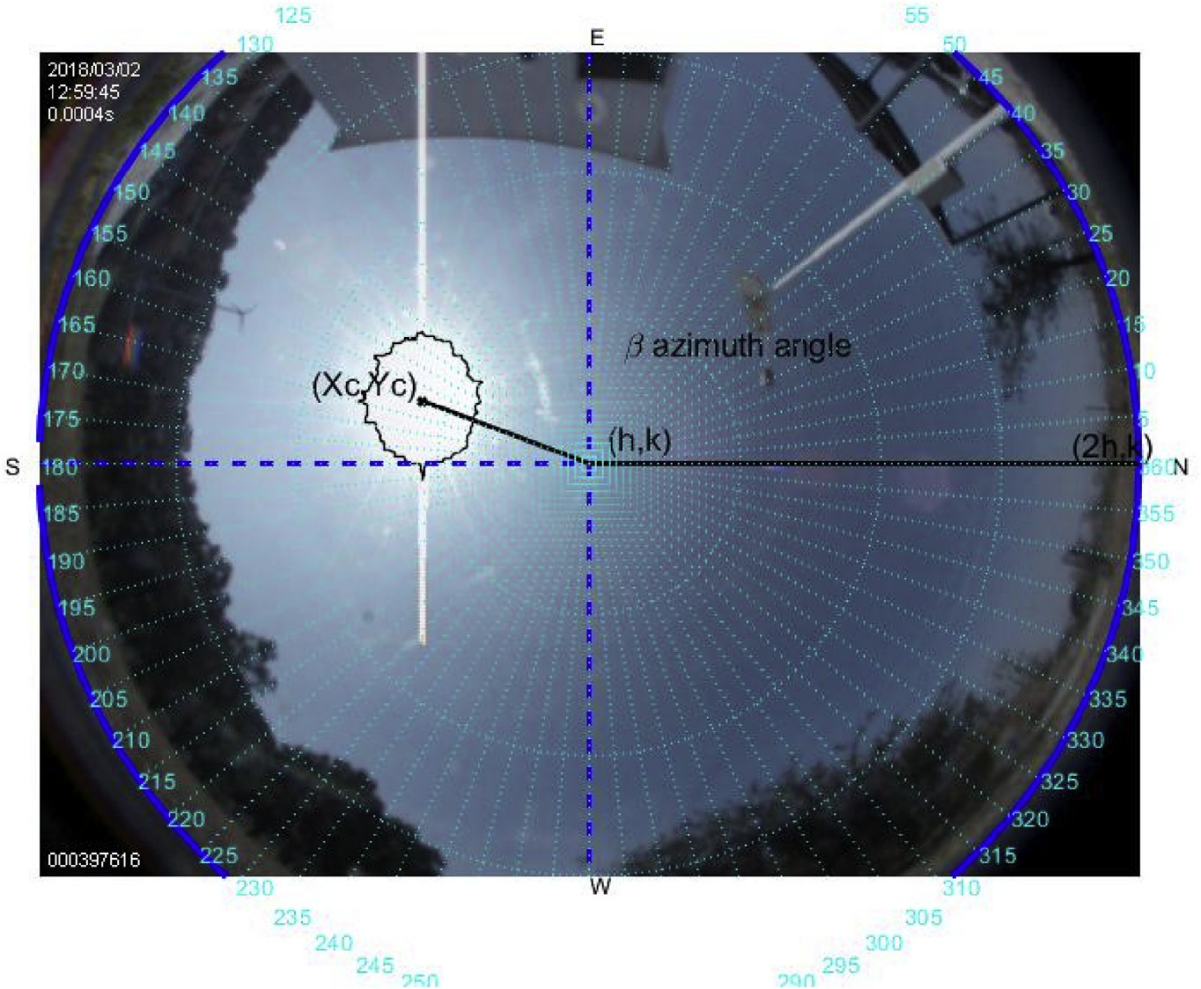
The image was taken at 12:59 hrs on March 2, 2018; the sun azimuth angle is 175.4° degrees and the solar elevation angle, 61.93°.

**Fig. 8:**
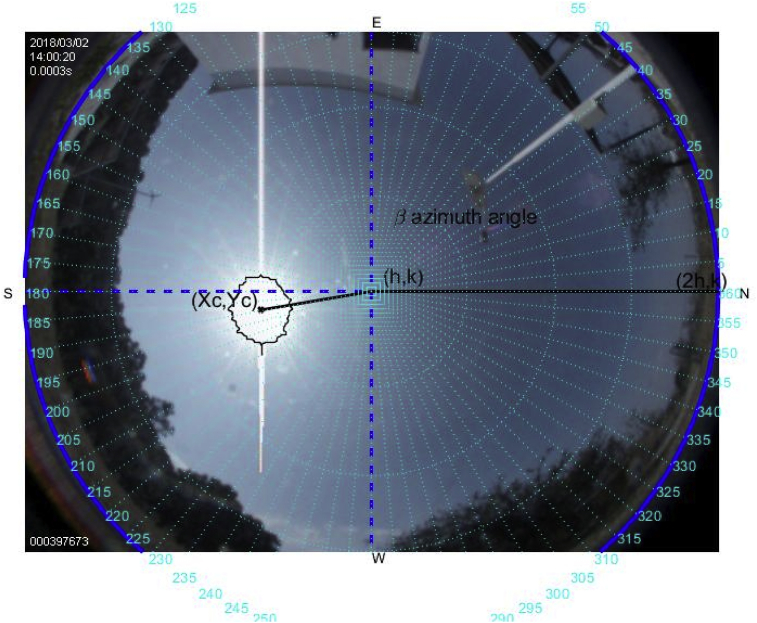
The image was taken at 14:00 hrs on March 2, 2018; the sun azimuth angle is 206.6° degrees and the solar elevation angle, 61.98°.

**Fig. 9:**
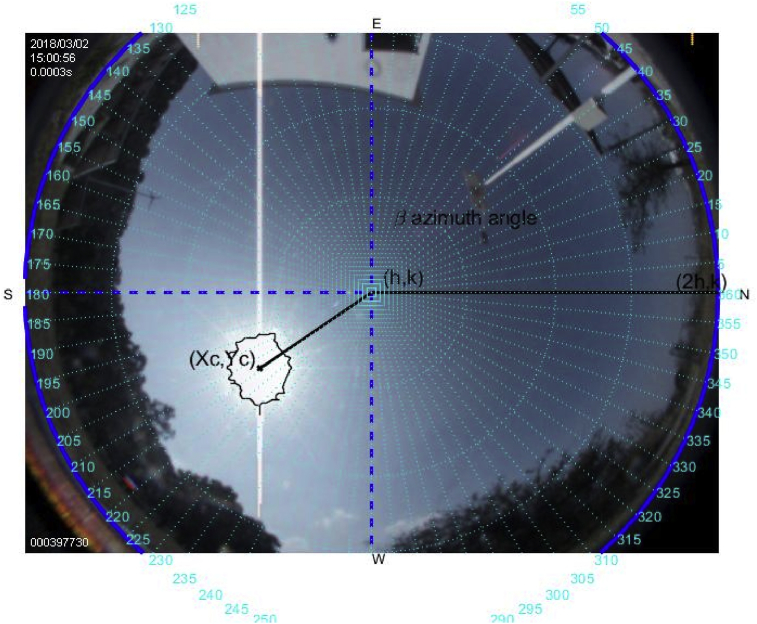
The image was taken at 15:00 hrs on March 2, 2018; the sun azimuth angle is 225.2° degrees and the solar elevation angle, 55.32°.

**Fig. 10:**
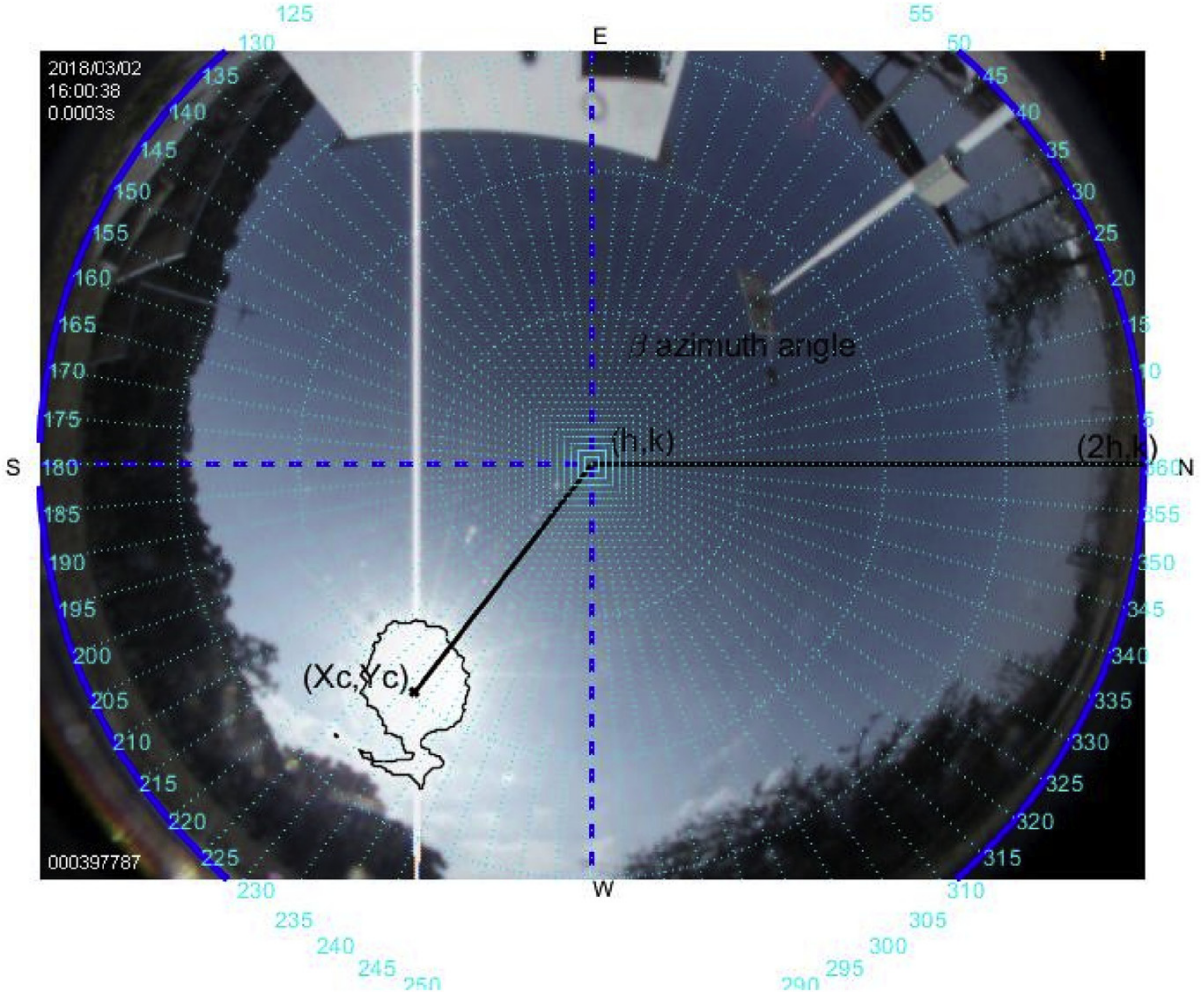
The image was taken at 16:00 hrs on March 2, 2018; the sun azimuth angle is 242.18° degrees and the solar elevation angle, 40.5°.

**Fig. 11:**
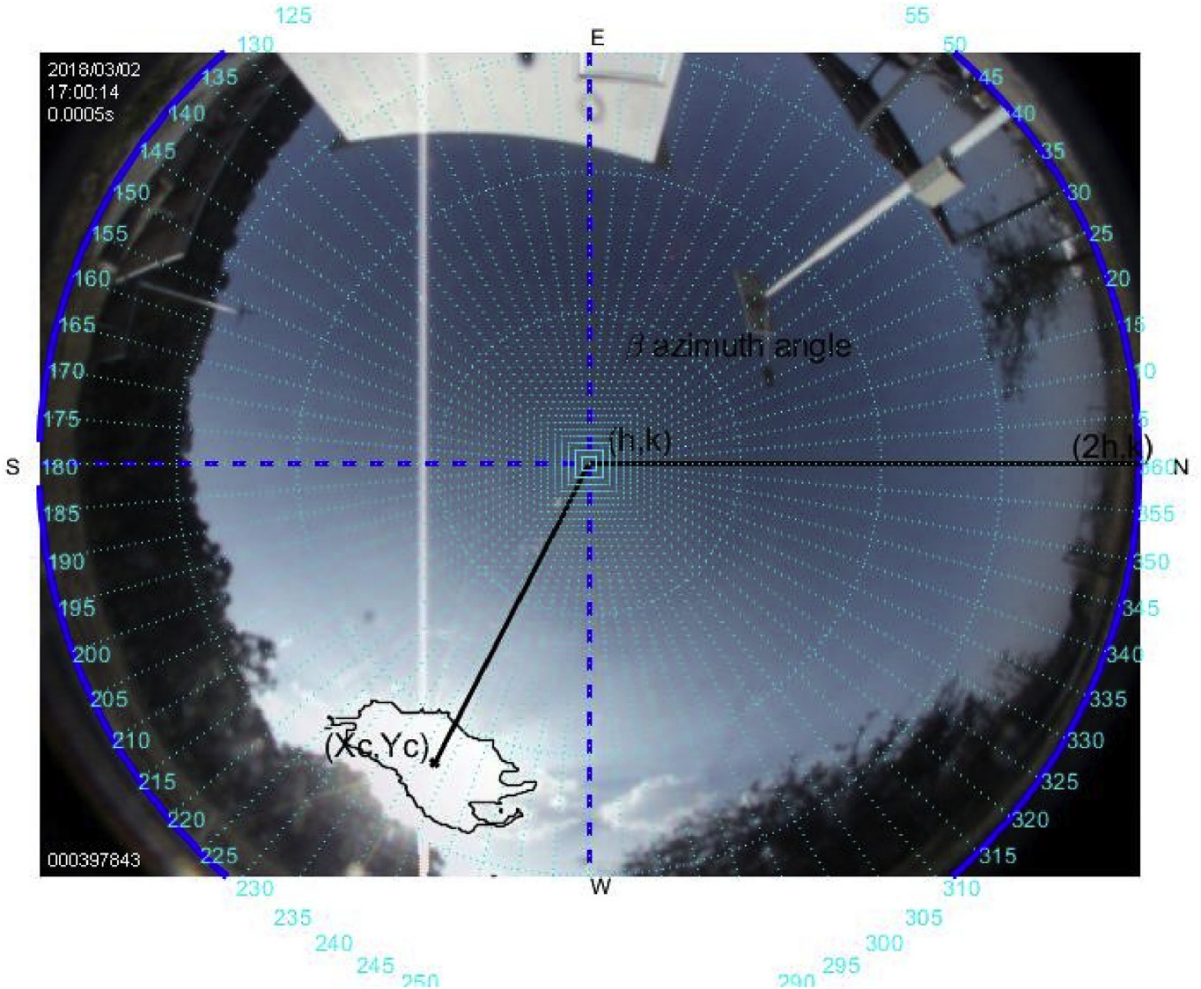
The image was taken at 17:00 hrs on March 2, 2018; the sun azimuth angle is 252.74° degrees and the solar elevation angle, 27.2°.

The tools above described are used to carry out this experiment on a sunny day on March 2, 2018. Figs. 6, 7, 8, 9, 10, and 11 exhibit results from the previous algorithm, where the sun azimuth and the elevation angles are estimated.

More than four hundred photographs were taken from 10:00 to 17:00 on March 2, 2018, in Guadalajara Jalisco Mexico allocated at 20°52′ latitude N and from 103°3’ longitude W and time zone -6, approximately every minute on a predominantly sunny day with some clouds. The sunrise, sunset and zenith information are summarized in Table 1. For brevity, only six photographs are shown hour by hour from 12:00 to 17:00. The images illustrate the results of the proposed method, Figs. 6, 7, 8, 9, 10, and 11.

**Table 1:** Sun path characteristic on March 2, 2018.

Parameters	Time	Position
Sunrise	7:11:52	96° 89′ from N
Sunset	18:58:29	263° from N
Solar noon	13:05:10	62° 85′ Elevation

Note that such Figures represent the estimation of the mentioned angles at different times of the day. The accuracy of the estimate has been very satisfactory when compared to the estimates provided by (NOAA) [27].

**Fig. 12:**
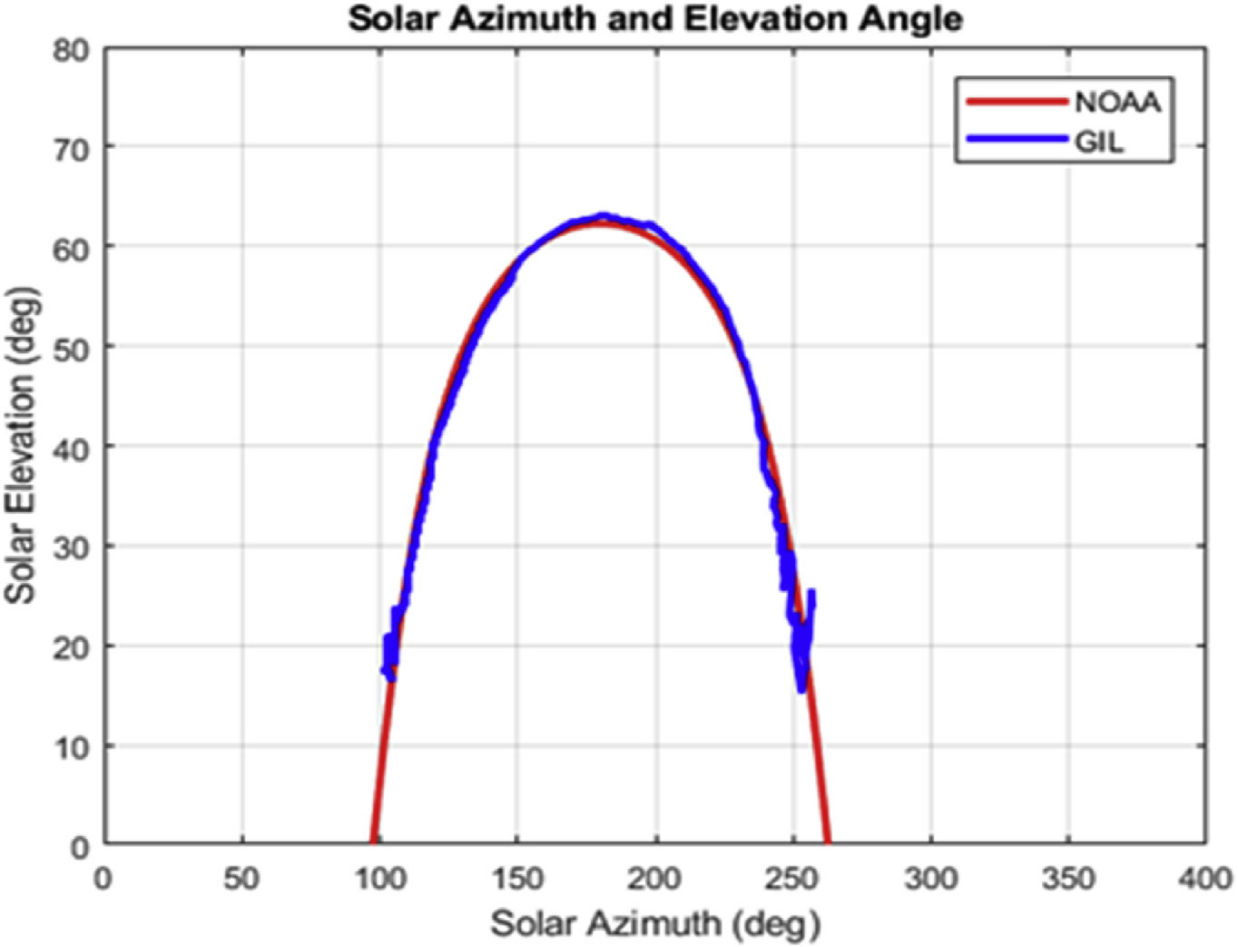
Graph of the sun azimuth and the elevation by NOAA vs the proposed method.

**Fig. 13:**
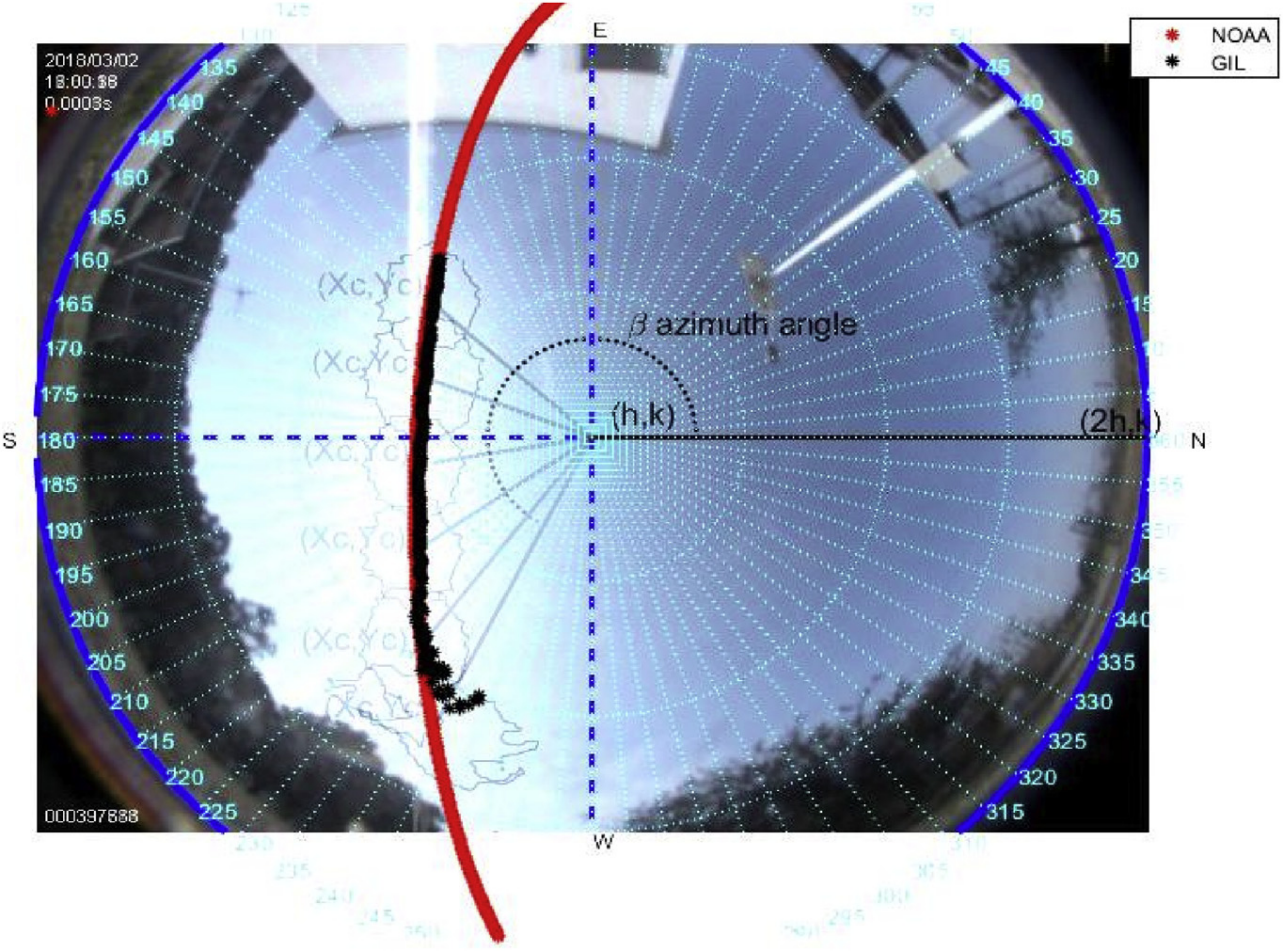
Trajectory by NOAA vs trajectory by the proposed method.

Figs. 12 and 13 depicts the sun azimuth and elevation angles estimated by the proposed strategy; also, such information estimated by NOAA is presented.

It is noteworthy that at the end of the trajectory taken by the proposed method (black) some estimations lie out the NOAA guide path (red). This is because the trajectory obeys the brightest point of the image that could be the periphery of a cloud, this does not rule out the effect of the atmosphere's mass of air [33]. Additionally, these data may not be considered, since the uptake of energy at that time in this hemisphere becomes scarce Fig. 13.

Table 2 describes the main differences among the NOAA estimates against the photoresist method and the proposed method. The proposed method is better in defining the azimuth angle but not in the solar elevation angle when making the error metric Mean Square Error (MSE).

**Table 2:** Comparison between the proposed method vs NOAA and LDRs method Figs. 7, 8, 9, 10, 11, and 12.

Hour	Az angle (NOAA)	Az angle (Fisheye)	Az angle (LDRs)	ElSun angle (NOAA)	ElSun angle (Fisheye)	ElSun angle (LDRs)
12:00	147.83°	148.32°	148.69°	58.4°	55.28°	56.67°
13:00	177.2°	175.4°	176.1°	62.8°	61.93°	60.87°
14:00	207.4°	206.6°	205.8°	59.8°	61.98°	61.12°
15:00	228.6°	225.2°	226.4°	50.6°	55.32°	53.56°
16:00	241.96°	242.18°	243.8°	39°	40.5°	41.34°
17:00	250.88°	252.74°	253.1°	26.18°	27.2°	27.87°
	**MSE**	1.22428571	1.40285714		1.91571429	1.71

AZ sun azimuth angle; ElSun elevation angle; LDRs Light Dependent Resistor.

To assess the differences between the estimated values and those found publicly, the following metrics for quantifying the error are proposed [34].

(i) The mean absolute deviation (MAD) is the sum of absolute differences between the actual value and the forecast divided by the number of observations.
(ii) The Mean square error (MSE) is probably the most commonly used error metric. It penalizes larger errors because squaring larger numbers has a greater impact than squaring smaller numbers. The MSE is the sum of the squared errors divided by the number of observations.
(iii) The root mean square error (RMSE) is the square root of the MSE.
(iv) The Mean Absolute Percentage Error (MAPE) is the average of absolute errors divided by actual observation values. Thus, the expressions (17)–(20) are used,

(17) $$MAD=\frac{\sum_{t=1}^{n}\left|A_{t}-F_{t}\right|}{n}$$

(18) $$MSE=\frac{{\sum_{t=1}^{n}\left(A_{t}-F_{t}\right)}^{2}}{n}$$

(19) $$RMSE=\sqrt{\frac{{\sum_{t=1}^{n}\left(A_{t}-F_{t}\right)}^{2}}{n}}$$

(20) $$MAPE=\frac{\sum_{t=1}^{n}\frac{\left|A_{t}-F_{t}\right|}{A_{t}}}{n}\;\times 100$$

where *A*_*t*_ is the data obtained from NOAA, *F*_*t*_ is the data obtained from the proposed method and *n* is the number of measurements. Table 3 summarizes the above indices for this application. Notice that the results are quite acceptable, except for some metrics.

**Table 3:** Metrics of error between the proposed method and NOOA.

Features	MAD	MSE	RMSE	MAPE
Error sun azimuth angle	1.13068423	3.47389284	1.8638382	0.49300272
Error elevation angle	0.45809665	5.8864384	2.43046606	4.97077973

### 4.2 Study case on August 11, 2018

**Fig. 14:**
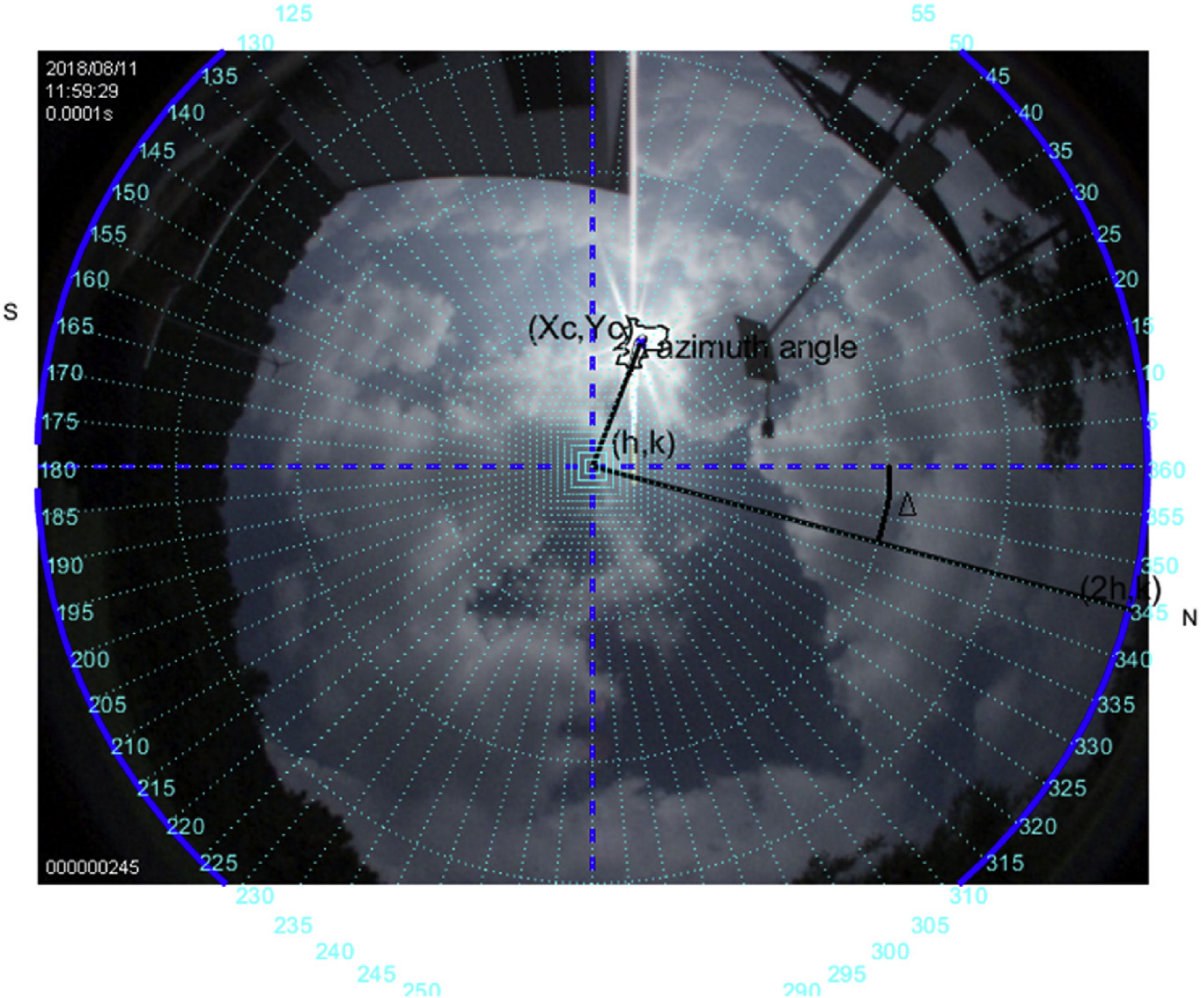
The image was taken at 11:59 hrs on August 11, 2018; the sun azimuth angle is 106.6°, and the elevation angle 75.7°.

**Fig. 15:**
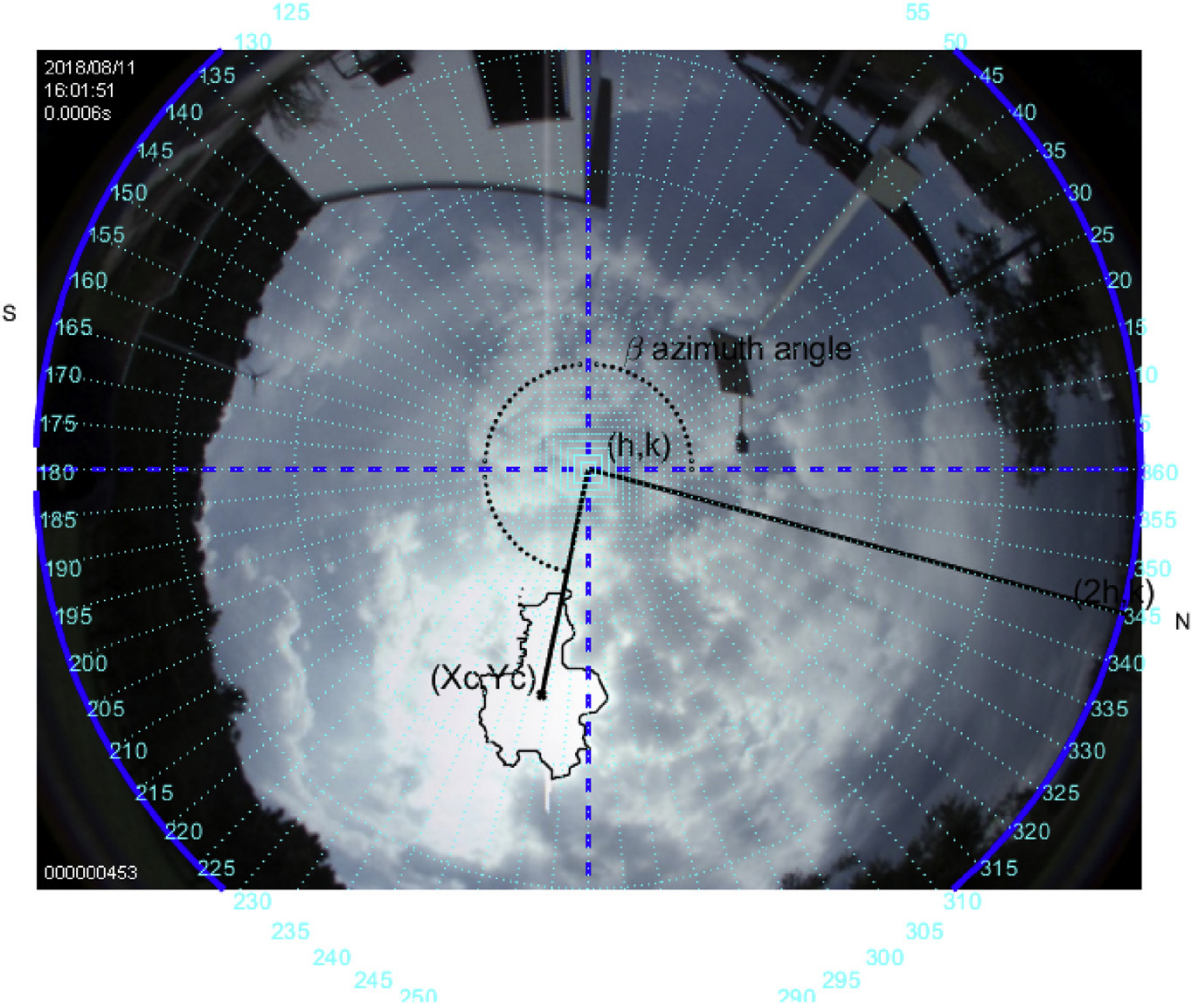
The image was taken at 16:01 hrs on August 11, 2018; the sun azimuth angle is 268.6°, and the elevation angle 49.1°.

**Fig. 16:**
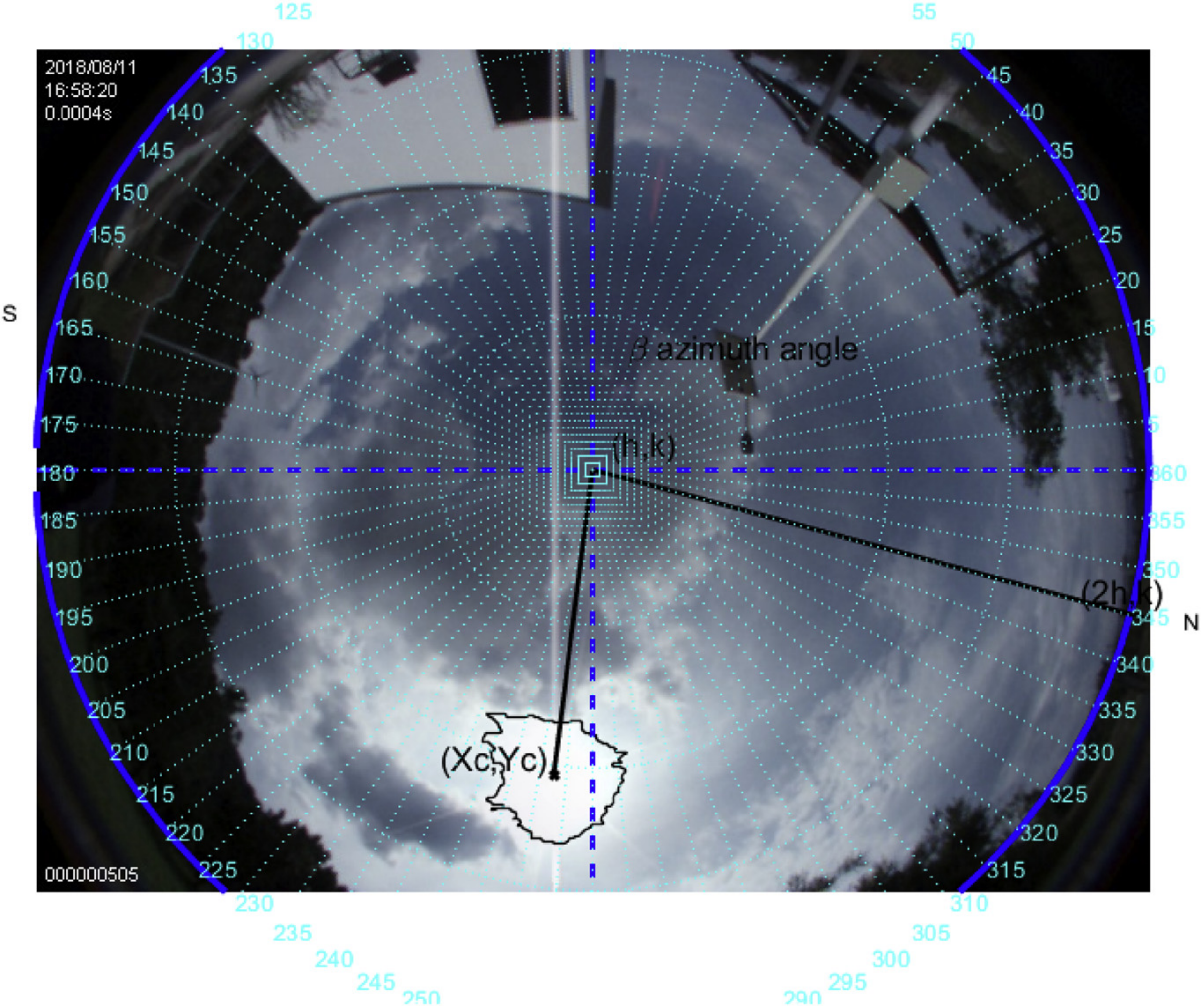
The image was taken at 16:58 hrs. on August 11, 2018; the sun azimuth angle is 272.6° and the elevation angle 36.2°.

Saturday, August 11 this day was cloudy with some intermittent rain; Figs. 14, 15, and 16 show the corresponding images.

This is a difficult case; however, it is shown that regardless of the sun's trajectory the algorithm will always follow the brightest point of the image in the field of view of the fish-eye camera. The metrics here do not coincide closely with those of the NOAA's due to the solar tracker follows the brightest point in the cloudy sky, which in such case adds uncertainty.

## 5 Discussion

Results shown in Table 2 are the comparison of the solar angles obtained by the proposed method and those obtained by NOOA; some minor discrepancies are observed. It should be noted that the proposed algorithm can allocate the solar tracker respect to the centroid of the brightest object obtained from the image, regardless of where the solar tracker is placed. This will always work in the same way given that the computational algorithm used is extremely easy to implement and with a robustness and acceptable precision since it does not require entering data such as latitude and longitude, time zone, summertime, date, or some control algorithm to adjust it to some trajectory. An additional advantage is that it does not require hardware such as GPS, photo-resistors, or sophisticated electronic equipment that will be affected quickly by inclement weather, for example, rain or heat. For this type of dual-axis solar tracker, only one fisheye camera is required, which makes completely achievable this model. Following are some characteristics of the equipment used.

Respect to the transmission mechanism, two gears of the following characteristics are used: the outer circumference is 10′-3/8″, and the smaller one is 9′-7/8″, with 80 teeth with a pitch of 1″, a tooth's height of 1″, and gear width of 1”. For calculating the solar elevation angle, only half the disc is used.

This experiment was carried out on a computer with a processor Intel(R) Core(TM) i7-2600k CPU@ 3.40 GHz with memory RAM 4 GB, 64 bits. Also, a fisheye cam SBIG AllSky-340 was used, which is perfect for monitoring observatory weather conditions. The KAI-0340 CCD sensor has 640 × 480 pixels at 7.4 microns square, and excellent sensitivity. Protected by an acrylic dome, to protect the included Fujinon FE185C046HA-1 lens has a 1.4 mm focal length at f/1.4 and provides excellent image quality all the way to the horizon.

The microcontroller ATmega2560 has 54 digital input/output pins (14 can be used as PWM outputs), 16 analog inputs, 4 UARTs (hardware serial ports), a 16 MHz crystal oscillator, a USB connection, a power jack, an ICSP header, and a reset button.

**Fig. 17:**
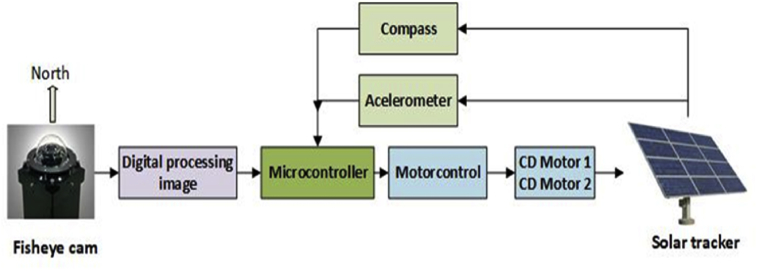
Solar tracker algorithm.

Fig. 17 is a graphic image that summarizes the solar tracker operation, which receives information from the fisheye camera. Digital treatment of images is applied to such information to calculate the solar angles. These angles are fed to a microcontroller that drives the motors. Simultaneously, the microcontroller receives data from a compass (which will give the data of the azimuth angle measured with the help of a step counter), and an accelerometer (which will give the data of the measured solar elevation) to reduce the error obtained in the scheme shown in Fig. 17.

Within a gray figure, the centroid is used as a strategy to allocate the point of greatest luminescence. The centroid is a geometric concept that depends on the symmetry of the object in question. The centroid of an object or figure can be defined as a fixed point of the isometry group of that figure. In this case, where it is ensured that the intensity of luminescence is the same concerning its neighboring pixels.

## 6 Conclusion

In this paper, an efficient method for solar tracking is proposed. It is based on the use of one type fisheye camera that captures sky images. After a careful image's treatment, it is possible to estimate the solar angles, which are used for correcting positioning photovoltaic panels to capture the greatest solar radiation. The proposition yields several advantages, such as simplicity, since it does not require the use of GPS or mathematical calculations and does not require the longitude or latitude information. However, it is quite important that the fisheye camera is oriented toward the north and that it is leveled concerning the ground. Several future works can be undertaken, for instance, the design and implementation of different maximum power point tracking. Results show that the proposition can attain quite good precision when compared with recognized algorithms.

## Declarations

### Author contribution statement

Juan M. Ramirez: Analyzed and interpreted the data; Wrote the paper.

Gerardo Garcia-Gil: Conceived and designed the experiments; Performed the experiments.

### Funding statement

This work was supported by Consejo Nacional de Ciencia y Tecnología. Juan M. Ramirez was supported by CONACyT – Red Tematica (project no. 294570).

### Competing interest statement

The authors declare no conflict of interest.

### Additional information

No additional information is available for this paper.
